# Mediating Effects of Emotion Regulation between Socio-Cognitive Mindfulness and Achievement Emotions in Nursing Students

**DOI:** 10.3390/healthcare9091238

**Published:** 2021-09-21

**Authors:** Mikyoung Lee, Keum-Seong Jang

**Affiliations:** 1 Department of Nursing, Kwangju Women’s University, Gwangju 62396, Korea; mikylee@kwu.ac.kr; 2 College of Nursing, Chonnam National University, Gwangju 61469, Korea

**Keywords:** nursing students, socio-cognitive mindfulness, emotion regulation, reappraisal, suppression, achievement emotions

## Abstract

Background: Mindfulness is known as an effective emotion regulation strategy and is beneficial for improving emotions. While meditative mindfulness has been widely studied, socio-cognitive mindfulness has received little attention in nursing literature, despite its potential benefits to the field. This study investigated relationships between nursing students’ socio-cognitive mindfulness, emotion regulation (reappraisal and suppression), and achievement emotions, and explored the mediating effects of emotion regulation. Methods: A total of 459 nursing students from three universities in Korea completed the questionnaire measuring the study variables. Structural equation modeling and path analysis were conducted to test the hypotheses. Results: Socio-cognitive mindfulness was found to positively influence reappraisal while negatively influencing suppression. Additionally, socio-cognitive mindfulness positively predicted positive achievement emotions but negatively predicted negative emotions. Reappraisal positively influenced positive emotions, whereas suppression positively influenced negative emotions. Furthermore, reappraisal mediated the link between mindfulness and positive emotions, and suppression mediated the link between mindfulness and negative emotions. Conclusions: Socio-cognitive mindfulness may be effective in regulating emotions among nursing students by enhancing reappraisal and reducing suppression. Mediating effects highlight the relevance of students’ emotion regulation in nursing education, suggesting the need to develop emotion regulation education programs.

## 1. Introduction

With the advent of the fourth industrial revolution, college students are required to possess socio-emotional skills that enhance the role of humans so as to counteract the impact of artificial intelligence [1]. In nursing education, cultivating students’ socio-emotional skills is particularly important because they constantly meet people in their future nursing practice. Therefore, nursing students should enhance their empathy, which is a representative socio-emotional skill [2]. To improve one’s empathy, students must first be competent in understanding their own emotions and managing those emotions effectively. However, nursing education continues to focus on delivering vast knowledge without much consideration of students’ emotions and emotion management. In particular, nursing students reportedly experience more stress and negative emotions from their heavy study load and practicum as compared to other majors [3]. This, coupled with the nature of the nursing profession, requires that nursing educators and researchers pay more attention to nursing students’ psychological well-being, management of emotions, and ability to empathize.

Mindfulness, which is known as an effective strategy for emotion regulation, focuses on the present moment and observes present experiences through wakefulness [4]. Mindfulness is categorized into two main theories: meditative mindfulness, defined by Kabat-Zinn [5]; and socio-cognitive mindfulness, defined by Langer [6]. Meditative mindfulness emphasizes a non-judgmental focus on the present through meditation, whereas socio-cognitive mindfulness underscores flexible contact with environments by improving one’s openness to external stimuli [7]. These conceptual distinctions between the two have been empirically evidenced in a few studies [8,9,10]; however, most studies on both mindfulness have been separately conducted. Countless studies exist on meditative mindfulness in many disciplines such as medicine [7,11], psychology [12,13,14], education [15,16,17], and nursing [18,19,20]. In comparison, research on socio-cognitive mindfulness exists in certain disciplines such as medicine [21] and psychology [22,23,24], and substantial studies have been conducted in the education field [8,25,26,27]. In the nursing field, however, socio-cognitive mindfulness has received little attention from researchers. 

According to Langer, mindfulness is defined as a process of forming new categories in active, open, and creative ways to the new information, which allows a person to consider situations from various perspectives [6]. Unlike meditative mindfulness, socio-cognitive mindfulness has demonstrated desirable effects after brief interventions, not including long-term meditation or training [8]. In addition, socio-cognitive mindfulness may be more adaptable to cognitive learning because it aims to enhance cognitive flexibility and performance [7]. Research has shown the benefits of socio-cognitive mindfulness in educational contexts. For example, students improved certain learning skills, such as their memory, concentration, problem-solving, and creativity, when they adopted more mindful attitudes and flexible thinking [26]. However, research on the effects of socio-cognitive mindfulness in nursing education is still in its early stages. Although limited, the existing studies of the effects of socio-cognitive mindfulness in nursing have highlighted the benefits to the field. For example, socio-cognitive mindfulness was positively related to nurses’ job satisfaction and personal accomplishment, whereas it was negatively related to emotional exhaustion and depersonalization [28]. Nursing students’ socio-cognitive mindfulness was positively associated with communication self-efficacy and empathy [29]. In addition, the socio-cognitive mindfulness program helped nurses cope with stress and emotional exhaustion more effectively; nurses were also better equipped to view their nursing environment and situations from various perspectives [30]. The most recent study found that socio-cognitive mindfulness was positively correlated with positive achievement emotions and academic outcomes while being negatively correlated with negative emotions [9]. This demonstrates that it is important to utilize socio-cognitive mindfulness in nursing education and practice. 

Emotion regulation is defined as the process influencing how and when individuals experience and express certain emotions, indicating one’s ability to manage emotional experiences and expressions [31]. Nurses encounter numerous work situations that require considerable emotional effort. They might often be emotionally exhausted while implementing nursing service. Therefore, it is important for them to apply effective strategies to manage their emotions properly, so that they can establish constructive relationships with patients and colleagues [32]. This will ultimately improve their psychological well-being, thereby allowing them to deliver higher quality nursing services. Indeed, effective strategies for emotion regulation could be one of the mandatory skillsets for nurses and pre-nurses. Gross proposed two main emotion regulation strategies: reappraisal and suppression [31]. The reappraisal strategy is a cognitive approach wherein someone can change how they feel by evaluating emotion-evoking situations. For example, if nurses pursue experiencing more positive emotions while they are providing nursing care, they can alter how they think about the situation [32]. Reappraisal occurs early in the emotion-generation process. Therefore, reappraisal can change the entire process of emotional experiences before emotions fully develop [33]. It may also be a useful strategy to reduce negative emotions but increase positive ones. In contrast, suppression is a type of emotional response that intends to suppress or hide emotions that have already been produced. Suppression takes place later in the emotion-generation process, and it can lower expressive behaviors without lessening the magnitude of negative emotions. Rather, suppression demands cognitive resources that deteriorate one’s memory capacity [31]. Previous literature on the relationship between mindfulness and emotion regulation has shown that meditative mindfulness is positively related to the reappraisal strategy and negatively related to the suppression strategy [34,35,36]. Socio-cognitive mindfulness also had a positive relationship with reappraisal [23] but a negative relationship with maladaptive strategies, including suppression and rumination [21].

Achievement emotions are defined as students’ specific emotions that are related directly to learning activities or academic outcomes [37]. Students’ emotions matter in the context of learning because students’ achievement emotions have a considerable influence on their learning quality and subsequent performance. Specifically, achievement emotions are strongly connected to students’ motivation, goal orientation, learning strategies, and psychological well-being [38] as well as self-regulation and emotion regulation [39]. While researchers have conducted numerous studies on achievement emotions in educational domains [39], little research on achievement emotions has been conducted in nursing. Research on nursing students’ emotions is highly warranted, given that they suffer from more stress and negative emotional experiences compared to students in other majors [3]. It has been reported that nursing students feel emotional difficulty during practicums when they face patients’ pain or death, or they feel helpless when they cannot offer sufficient professional expertise in clinical situations [40]. If such an unfavorable situation is not managed well, it may have detrimental effects on nursing students’ learning outcomes and psychological well-being, which might negatively influence their future nursing practice. Previous studies have found that socio-cognitive mindfulness was positively associated with positive emotions [23] and negatively associated with negative emotions [7,21,23,24]. Regarding the relationships between emotion regulation strategies and emotions, reappraisal had a positive relationship with positive emotions, whereas suppression had a positive relationship with negative emotions [32,33,34,41]. However, there is little empirical evidence regarding these relationships among nurses and nursing students, even though regulating emotions is increasingly essential in their workplace as compared to other professionals. 

The aforementioned studies on the relationships that socio-cognitive mindfulness has with emotion regulation or emotions have been conducted with adults in general situations. Some studies were conducted with college students in educational contexts, but they hardly included nursing students. In addition, these studies predominantly examined negative emotions when examining socio-cognitive mindfulness’s effects on emotions. Therefore, to fill these research gaps, this study integrates the under-examined socio-cognitive mindfulness and achievement emotions into the nursing field. This study aims to investigate the relationships between socio-cognitive mindfulness, emotion regulation, and both positive and negative achievement emotions among nursing college students. This study further explores the mediating effect of emotion regulation between socio-cognitive mindfulness and achievement emotions. Based on theoretical backgrounds and the literature discussed above, the following hypotheses are proposed:

**Hypothesis** **1.** **(H1)** *Socio-cognitive mindfulness correlates positively with reappraisal but negatively with suppression.*

**Hypothesis** **2.** **(H2)** *Socio-cognitive mindfulness correlates positively with positive achievement emotions but negatively with negative achievement emotions*.

**Hypothesis** **3.** **(H3)** *Reappraisal is positively associated with positive achievement emotions*.

**Hypothesis** **4.** **(H4)** *Suppression is positively associated with negative achievement emotions*.

**Hypothesis** **5.** **(H5)** *Reappraisal mediates therelationship between socio-cognitive mindfulness and positive emotions*.

**Hypothesis** **6.** **(H6)** *Suppression mediates therelationship between socio-cognitive mindfulness and negative emotions*.

## 2. Materials and Methods

### 2.1. Research Design

We designed a cross-sectional study to test these hypotheses. This allowed us to examine the hypothesized relationships among the study variables as well as the mediating effect of emotion regulation between socio-cognitive mindfulness and achievement emotions.

### 2.2. Participants and Procedure

The participants were 459 nursing students (age M = 21.39, SD = 1.61, 87.6% female), consisting of 210 sophomores, 156 juniors, and 93 seniors from three universities in one metropolitan city in South Korea. To decide the sample size, we considered Yu’s suggestion that the number of 200–400 would be desirable when utilizing a maximum likelihood in structural equation modeling (SEM) [42]. We also considered the number of study variables. Furthermore, the use of latent variables instead of observed variables for analysis in this study affected the number of participants that we approached. In particular, Kline [43] recommended a sample size of 5 to 20 times the total number of variables to conduct SEM effectively. Therefore, we believe that a sample size of 459 nursing students was sufficient for our research model. Freshmen were excluded from our participant pool as nursing students begin taking major-specific classes during their sophomore year. As we were examining students’ achievement emotions regarding nursing major classes, students who were not yet enrolled in nursing major classes were not considered for our participant pool. 

We collected data from 11 December 2019 until 7 January 2020. We received ethical approval from the Institutional Review Board at C University (1040198-191018-HR-110-02). Approvals from each nursing department Dean at the three universities were also obtained. Our questionnaire consisted of nursing students’ socio-cognitive mindfulness, emotion regulation, and achievement emotion measures. Background information on age, gender, grade, and academic outcomes was also collected. The first author, who was not related to the participants, described the purpose and significance of the research and collected data. The participants were assured that their responses would remain confidential and only be used for research purposes. The questionnaire was distributed during a break; the students voluntarily signed a written consent form and completed the questionnaire within 20 min.

### 2.3. Measures

#### 2.3.1. Socio-Cognitive Mindfulness

The Korean validated version of the Langer Mindfulness Scale (LMS) [44] developed by Bodner and Langer [45] was used to assess participants’ socio-cognitive mindfulness. The LMS measures four components of socio-cognitive mindfulness with 21 items: novelty seeking (six items), novelty producing (six items), flexibility (four items), and engagement (five items). Sample items include: “I like to figure out how things work” (novelty seeking), “I am very creative” (novelty producing), “I can behave in many different ways for a given situation” (flexibility), and “I attend to the big picture” (engagement). Responses to all the items were assessed on a five-point response scale ranging from strongly disagree (1) to strongly agree (5). Cronbach’s alphas were acceptable at 0.73, 0.86, 0.69, and 0.71 for novelty seeking, novelty producing, flexibility, and engagement, respectively.

#### 2.3.2. Emotion Regulation

The Emotion Regulation Questionnaire (ERQ) [33] is extensively employed to assess emotion regulation strategies. This study used the Korean version of the ERQ [46] to evaluate nursing students’ emotion regulation. The Korean ERQ evaluates two strategies of emotion regulation: reappraisal with six items (e.g., “I control my emotions by changing the way I think about the situation I’m in”) and suppression with four items (e.g., “I keep my emotions to myself”). Participants answered using a scale between strongly disagree (1) and strongly agree (5). Cronbach’s alpha was 0.83 for reappraisal and 0.77 for suppression, demonstrating good internal consistency. 

#### 2.3.3. Achievement Emotions 

The Korean Achievement Emotions Questionnaire (AEQ) [47] version originally developed by Pekrun et al. [48] was selected to evaluate achievement emotions. For participants of this study, the existing AEQ, which is a modified version of the Korean AEQ for Korean nursing students [2], was used. This instrument assesses three positive achievement emotions (enjoyment, hope, pride) with 12 items and five negative emotions (boredom, anger, anxiety, hopelessness, and shame) with 20 items. Sample items include “I enjoy my class” (positive emotion) and “I get annoyed during my class” (negative emotions). A five-point answer scale was utilized between strongly disagree (1) and strongly agree (5). This measure displayed good internal consistency with a Cronbach’s alpha of 0.85 for positive emotions and 0.91 for negative emotions. 

### 2.4. Data Analyses

Correlations and means of the variables were analyzed with SPSS 25 program. We implemented SEM using Mplus 7 program [49] to test the hypotheses of the relationships between the variables. We also conducted path analysis with Mplus 7 to investigate the mediating effects of emotion regulation. We used this program for analysis because it is a powerful analytical software with some benefits when performing SEM. For example, it presents model information to verify whether the model suits the data properly or not, utilizes the full information maximum likelihood method to deal with missing data, and corrects measurement error [49].

## 3. Results

### 3.1. Preliminary Results

Table 1 exhibits correlations and means of the variables. The means of nursing students’ socio-cognitive mindfulness were higher than 3 out of 5, with engagement found to be the highest (M = 3.66, SD = 0.59) and flexibility reported as the lowest (*M* = 3.05, *SD* = 0.55). In terms of emotion regulation, participants reported higher reappraisal (*M* = 3.49, *SD* = 0.65) than suppression (*M* = 2.73, *SD* = 0.83). They also expressed more positive achievement emotions (*M* = 3.09, *SD* = 0.56) than negative emotions (*M* = 2.82, *SD* = 0.67). 

All sub-categories of socio-cognitive mindfulness correlated positively with reappraisal but not with suppression, except for a negative correlation with engagement. The sub-categories were also positively associated with positive emotions and negatively associated with negative emotions. Furthermore, reappraisal was positively linked to positive emotions but negatively linked to negative emotions, whereas suppression was positively linked to negative emotions but not linked to positive emotions. 

### 3.2. Relationships between Socio-Cognitive Mindfulness, Emotion Regulation, and Achievement Emotions (Hypotheses 1–4)

To examine the relationships between nursing students’ socio-cognitive mindfulness, emotion regulation, and achievement emotions, we performed SEM with Mplus 7 [49]. In the original model, correlations among the four sub-categories of socio-cognitive mindfulness were contemplated. In addition, paths from each mindfulness category to each emotion regulation strategy and achievement emotions, as well as from each emotion regulation strategy to achievement emotions, were added. We evaluated the model with the chi-square (χ^2^), comparative fit index (CFI), root-mean-square-error of approximation (RMSEA), Tucker-Lewis Index (TLI), and standardized root-mean-square-residual (SRMR). The model fit was acceptable considering the standard of CFI > 0.90, TLI > 0.90 [50], and RMSEA < 0.080 and SRMR < 0.080 [51], with χ^2^ (1857) = 5142.805, *p* < 0.001, CFI = 0.901, TLI = 0.907, RMSEA = 0.059, and SRMR = 0.075.

**Figure 1:**
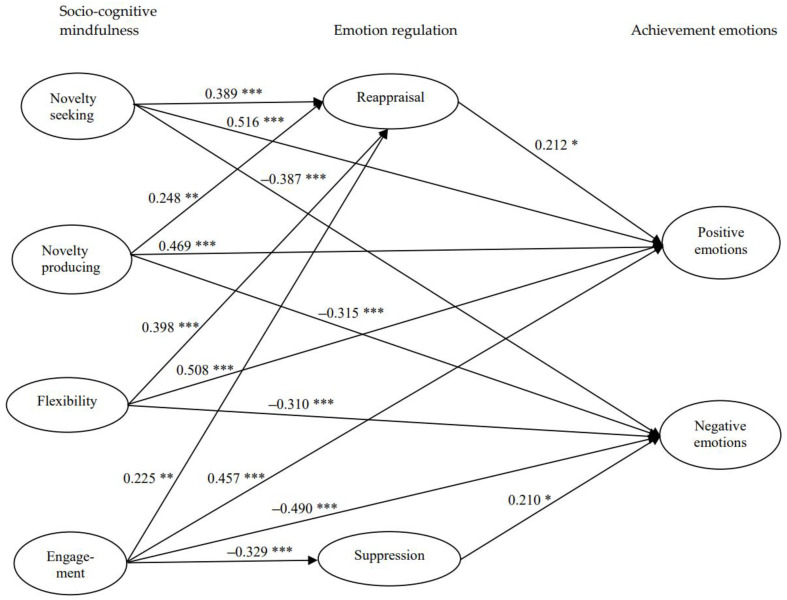
Structural parameter estimates for effects of socio-cognitive mindfulness on nursing students’ emotion regulation and achievement emotions. Only significant coefficients are displayed. * *p* < 0.05. ** *p* < 0.01. *** *p* < 0.001.

The significant path coefficients among socio-cognitive mindfulness, emotion regulation, and achievement emotions are displayed in Figure 1. First, regarding the relationship between socio-cognitive mindfulness and emotion regulation (Hypothesis 1), socio-cognitive mindfulness correlated positively with reappraisal but negatively with suppression. Specifically, all categories of novelty seeking (β = 0.389, *p* < 0.001), novelty producing (β = 0.248, *p* < 0.01), flexibility (β = 0.398, *p* < 0.001), and engagement (β = 0.225, *p* < 0.01) positively influenced reappraisal. Additionally, engagement was the one category (β = −0.329, *p* < 0.001) that negatively influenced suppression. 

Second, concerning the relationship between socio-cognitive mindfulness and achievement emotions (Hypothesis 2), socio-cognitive mindfulness was positively related to positive achievement emotions while being negatively related to negative achievement emotions. Novelty seeking (β = 0.516, *p* < 0.001), novelty producing (β = 0.469, *p* < 0.001), flexibility (β = 0.508, *p* < 0.001), and engagement (β = 0.457, *p* < 0.001) positively influenced positive emotions. Meanwhile, novelty seeking (β = −0.387, *p* < 0.001), novelty producing (β = −0.315, *p* < 0.001), flexibility (β = −0.310, *p* < 0.001), and engagement (β = −0.490, *p* < 0.001) negatively influenced negative emotions. 

Third, regarding the relationship between emotion regulation and achievement emotions (Hypotheses 3 and 4), reappraisal was positively associated with positive emotions (β = 0.212, *p* < 0.05). Suppression was positively associated with negative emotions (β = 0.210, *p* < 0.05). 

### 3.3. Mediating Effects of Emotion Regulation (Hypotheses 5 and 6)

To inspect the mediating effects of emotion regulation on the relationship between nursing students’ socio-cognitive mindfulness and achievement emotions, path analysis was conducted considering the significant links between the variables. Mediation analysis supported the hypothesis that reappraisal mediates the links between socio-cognitive mindfulness and positive achievement emotions. The analysis also indicated that suppression had a mediating effect on the relationship between socio-cognitive mindfulness and negative achievement emotions. Table 2 displays the detailed mediating effects between the variables. 

In the relationship between novelty seeking, reappraisal, and positive achievement emotions, novelty seeking correlated positively with reappraisal (a = 0.411, *p* < 0.001), and reappraisal correlated positively with positive achievement emotions (b = 0.208, *p* < 0.01). The direct effect of novelty seeking on positive achievement emotions was lowered after controlling for the reappraisal effect (c’= 0.444, *p* < 0.001), in comparison with the total effect (c = 0.529, *p* < 0.001). The indirect effect (mediating effect) by virtue of reappraisal was significant (a × b = 0.085, *p* < 0.01). This implies that reappraisal partially mediates the link between novelty seeking and positive emotions. In the relationship between novelty producing, reappraisal, and positive achievement emotions, novelty producing correlated positively with reappraisal (a = 0.251, *p* < 0.01), and reappraisal correlated positively with positive emotions (b = 0.207, *p* < 0.01). The direct effect of novelty producing on positive emotions was attenuated after controlling for the reappraisal effect (c’= 0.459, *p* < 0.001). The mediating effect via reappraisal was significant (a × b = 0.052, *p* < 0.01), suggesting that reappraisal partially mediates the relationship between novelty producing and positive emotions.

In the relationship between flexibility, reappraisal, and positive achievement emotions, flexibility correlated positively with reappraisal (a = 0.432, *p* < 0.001), and reappraisal correlated positively with positive emotions (b = 0.211, *p* < 0.01). The direct effect of flexibility on positive emotions was weakened after controlling for the reappraisal effect (c’= 0.441, *p* < 0.001). The mediating effect by reappraisal was notable (a × b = 0.091, *p* < 0.01), implying that reappraisal partially mediates the association between flexibility and positive emotions. In the relationship between engagement, reappraisal, and positive achievement emotions, engagement correlated positively with reappraisal (a = 0.227, *p* < 0.01), and reappraisal correlated positively with positive emotions (b = 0.233, *p* < 0.01). The direct effect of engagement on positive emotions was lowered after controlling for the reappraisal effect (c’= 0.409, *p* < 0.001). The mediating effect by reappraisal was substantial (a × b = 0.053, *p* < 0.01), indicating that reappraisal partially mediates the link between engagement and positive emotions.

Finally, in the relationship between engagement, suppression, and negative achievement emotions, engagement correlated negatively with suppression (a = −0.298, *p* < 0.001), and suppression correlated positively with negative emotions (b = 0.216, *p* < 0.01). The direct effect of engagement on negative emotions was attenuated after controlling for the suppression effect (c’= −0.431, *p* < 0.01). The indirect effect via suppression was substantial (a × b = −0.064, *p* < 0.01), revealing that suppression partially mediates the association between engagement and negative emotions.

## 4. Discussion 

Despite abundant studies on meditative mindfulness, investigation of socio-cognitive mindfulness is limited, especially in nursing. Additionally, previous emotion studies in nursing concentrated on students’ general affect rather than specific emotions in the learning context. Therefore, we aimed to fill this research gap by investigating the relationships between socio-cognitive mindfulness, emotion regulation, and achievement emotions, as well as the mediating effects of emotion regulation among nursing students.

### 4.1. Relationships between Socio-Cognitive Mindfulness, Emotion Regulation, and Achievement Emotions 

First, our findings generally supported Hypothesis 1 that socio-cognitive mindfulness positively influences reappraisal while negatively influencing suppression. This result corroborates previous findings that maladaptive emotion regulation strategies are reduced through socio-cognitive mindfulness [21], and college students’ socio-cognitive mindfulness correlated positively with reappraisal [23]. Socio-cognitive mindfulness promotes reappraisal by considering environments with multiple perspectives, thus changing thoughts in desirable directions while preventing suppression [22]. This statement reflects that the main characteristics of socio-cognitive mindfulness are open-mindedness to new information, the establishment of new distinctions, and a concentration on the present considering contextual situations [52]. In addition, nursing students equipped with socio-cognitive mindfulness can evaluate new information from different viewpoints. This allows for increased thinking ability that promotes flexibility and novel insights. Going forward, nurses could employ reappraisal in adverse situations. 

Looking into the links between the four sub-categories of socio-cognitive mindfulness and emotion regulation, suppression was influenced only by engagement. Given that the relationships between the four sub-categories and emotion regulation are under-examined, it is limited to provide a deeper discussion of their non-significant results with suppression. One possible reason may be the positive correlation between reappraisal and suppression in the present study (*r* = 0.19, *p* < 0.001). As previous research has also found a positive relationship between these strategies, this explains that both strategies possess self-capability to down-regulate emotions; however, they differ in processing cognitive tactics [32]. The present result still leaves unanswered questions. Future research is necessary to explore the relationships between socio-cognitive mindfulness sub-categories and emotion regulation or emotional experiences in various, non-academic settings. 

Second, socio-cognitive mindfulness positively predicted nursing students’ positive achievement emotions but negatively predicted negative emotions, supporting Hypothesis 2. This finding is concordant with previous findings that socio-cognitive mindfulness interventions enabled college students to enhance their interest in learning by categorizing new distinctions in creative ways; it also helped students experience positive emotions by inducing immersion through cognitive flexibility [26,53,54]. This result reinforces that nursing students’ socio-cognitive mindfulness positively influenced their thinking ability and creativity. Consequently, this encourages positive emotions while discouraging negative ones. For example, nurses’ mindfulness increased positive psychological attributes, such as job satisfaction and personal accomplishment, but reduced negative attributes, such as stress or emotional exhaustion [28,30]. Additionally, nursing students improved their emotional experiences by generating higher communication self-efficacy and empathy through socio-cognitive mindfulness [29]. This finding indicates that students with higher mindfulness may try harder to achieve insight and produce novel problem-solving alternatives. Their increased insight and creative thinking induce positive emotions by allowing for more flexible and open-minded, thus expanding cognitive performance [26]. 

Finally, reappraisal positively predicted nursing students’ positive achievement emotions while suppression positively predicted negative emotions, supporting Hypotheses 3 and 4. This result is consonant with Gross and John [33], who verified that individuals applying reappraisal experienced more positive emotions, whereas individuals employing suppression experienced more negative emotions. This confirmation is also supported by a recent nursing study [32] that demonstrated that nurses’ reappraisal was positively related to positive emotions, while suppression was positively related to negative emotions—also in line with several studies [34,55]. Therefore, reappraisal is a predictor of pleasant emotional experiences, leading to an increase in emotionally rewarding situations; accordingly, reappraisal plays a key role in increasing positive emotions [34]. Furthermore, reappraisal modifies inner feelings and expressions by intervening in the emotion generation process before emotions develop [9]. Based on the assumptions in Gross and John [33], nursing students applying reappraisal could view and re-evaluate learning environments optimistically even in stressful academic situations. This increases positive emotions and decreases negative emotions. In contrast, suppression only revises people’s expressive behaviors; in actuality, suppression occurs after emotions have already developed, thereby wasting cognitive energy [31]. Students using suppression may not effectively recover negative emotions and continuously experience them because they handle stressful learning environments by suppressing or hiding their inner feelings [33]. In summary, the present findings emphasize that reappraisal may be beneficial for nursing students’ emotional experiences by promoting positive emotions, whereas suppression is disadvantageous as it facilitates negative emotions. 

### 4.2. Mediating Effects of Emotion Regulation

The mediating effects of emotion regulation on the relationships between nursing students’ socio-cognitive mindfulness and achievement emotions were approved, supporting Hypotheses 5 and 6 overall. Specifically, reappraisal mediated the links between novelty seeking/novelty producing/flexibility/engagement and positive achievement emotions. Meanwhile, suppression mediated the link between engagement and negative achievement emotions. This mediation indicates the direct influence of socio-cognitive mindfulness as well as the indirect influence of reappraisal on positive emotions; in addition, socio-cognitive mindfulness directly influences negative emotions, and suppression indirectly influences them as well. That is, students with higher mindfulness may apply reappraisal more frequently, thereby experiencing more positive emotions, but use suppression less, thereby feeling fewer negative emotions. 

The mediating effects elucidate that socio-cognitive mindfulness encourages reappraisal but inhibits suppression, ultimately improving students’ positive emotions while reducing negative ones. This finding emphasizes the benefits of reappraisal and the harm of suppression in the relationship between students’ mindfulness and achievement emotions. Although future studies need to explore the mediating roles of emotion regulation in more detail, this result implies that students’ emotion regulation could be one mechanism for explaining the relationship between nursing students’ socio-cognitive mindfulness and achievement emotions. This implication highlights the relevance of students’ emotion regulation in the context of nursing education. 

### 4.3. Implications and Limitations

This research examines Langer’s socio-cognitive mindfulness in the field of nursing research, a previously neglected area. This is the first investigation of the influence that socio-cognitive mindfulness has on nursing students’ emotion regulation and achievement emotions. Considering innumerable studies on meditative mindfulness but scarce research on socio-cognitive mindfulness in the nursing field, this study pioneers this research area. Furthermore, this study broadens emotion study in nursing by exploring students’ achievement emotions in educational contexts, given that previous studies focused on nursing students’ general affect. This study facilitates a better understanding of students’ learning emotions. Therefore, it provides scientific knowledge about nursing studies and contributes to the practical improvement of nursing education by offering a basis for developing educational interventions. 

Regarding emotion management research in nursing, researchers have paid significant attention to the emotional labor framework of Hochschild [56] with surface and deep acting [10]. However, the emotion regulation framework of Gross [9] with reappraisal and suppression has been understudied. The present study is rare in that it utilized Gross’s [9] framework. In fact, this is the earliest study that identifies the mediating role of emotion regulation between nursing students’ socio-cognitive mindfulness and achievement emotions. Our discovery of the mediating effect stresses the need to develop emotion regulation education programs to enhance nursing students’ overall well-being. This result also suggests that including emotion regulation-related classes in the nursing curriculum will benefit future nurses. 

This study has some limitations. As the data was self-reported by participants, there may be bias inherent to the results. Generally, people tend to express socially desirable responses when asked, meaning that when using self-reported data, there is always a chance of obtaining biased results. To complement this drawback, future research should use both self-reported data and third-party data from peers or educators for a more accurate and objective measurement. Qualitative research would be beneficial for a more in-depth understanding of students. Another limitation is that the results are based on cross-sectional data; this causes difficulty in guaranteeing a complete framework with our variables. Given the nature of cross-sectional research, the causal relationships between variables should be carefully interpreted. Future studies should conduct longitudinal research to overcome this limitation. Finally, previous literature has demonstrated the effectiveness of mindfulness-based cognitive behavioral therapy in treating health issues [57,58,59]. Therefore, it would be worthwhile to integrate cognitive behavioral therapy and socio-cognitive mindfulness in future research. 

## 5. Conclusions

We found that socio-cognitive mindfulness may be effective in regulating emotions among nursing students as it promotes reappraisal and inhibits suppression. Socio-cognitive mindfulness also allows nursing students to experience more positive emotions but less negative emotions. This is because it facilitates more flexible and open-minded thinking, thereby enhancing cognitive performance, learning skills, and creativity. Furthermore, reappraisal may be beneficial to students’ emotional experiences as it increases positive achievement emotions. Suppression may be detrimental to students’ emotional well-being as it generates more negative emotions. Notably, the mediating effect of emotion regulation emphasizes the advantages of reappraisal and the disadvantages of suppression for nursing students. 

For the future, we suggest that nursing educators expand research on nursing students’ emotional experiences and emotion regulation to enhance the quality of college life for nursing students, with an emphasis on improving their psychological well-being. Based on the present results, the education system for nurses would benefit immensely from the development of a systematic training program for students’ emotion regulation. If nursing educators were to design, implement, and evaluate the effects of the program, there is much to be gained for the students and the overall profession. Through the emotion regulation program, nursing students would be able to first recognize their emotions, and then better understand others’ emotions. This might have the positive effect of improving nurses’ empathy, which is a crucial ability. Next, we suggest applying socio-cognitive mindfulness to nursing students and verifying the positive impacts. It would be beneficial to develop a program based on socio-cognitive mindfulness and implement it in nursing schools. More active research on this under-examined mindfulness is strongly recommended. Finally, we propose integrating classes related to mindfulness and emotion regulation into the nursing curriculum. Hopefully, this would lead to a long-term curricular change. If students can cultivate mindful attitudes in class, they will improve their self-awareness and learn about effective self-management. This will enable them to better concentrate on their studies. More importantly, nursing students will have the opportunity to acquire and practice mindfulness and emotion regulation strategies, which are lifelong skills required that facilitate better nursing and an improved quality of life. 

## Figures and Tables

**Table 1:** Correlations, Means, and Standard Deviations for the Study Variables (*N* = 459).

Variables		1	2	3	4	5	6	7	8
Socio-cognitive mindfulness	1. Novelty seeking	1							
	2. Novelty producing	0.70 ***	1						
	3. Flexibility	0.65 ***	0.69 ***	1					
	4. Engagement	0.52 ***	0.39 ***	0.34 ***	1				
Emotion regulation	5. Reappraisal	0.33 ***	0.24 ***	0.27 ***	0.25 ***	1			
	6. Suppression	−0.05	−0.07	0.03	−0.14 **	0.19 ***	1		
Achievement emotions	7. Positive emotions	0.49 ***	0.47 ***	0.37 ***	0.43 ***	0.29 ***	−0.03	1	
	8. Negative emotions	−0.37 ***	−0.31 ***	−0.26 ***	−0.47 ***	−0.14 **	0.17 ***	−0.55 ***	1
	Mean ^a^	3.41	3.10	3.05	3.66	3.49	2.73	3.09	2.82
	SD	0.61	0.72	0.55	0.59	0.65	0.83	0.56	0.67

Note. ^a^ Possible range 1–5. ** *p* < 0.01. *** *p* < 0.001.

**Table 2:** Indirect Effects of Socio-cognitive Mindfulness on Achievement Emotions through Emotion Regulation: Path Analysis (*N* = 459).

IV	M	DV			Total Effect	Direct Effect	Indirect Effect
			IV→M (a)	M→DV (b)	IV→DV (c)	IV→DV (c’)	IV→M→DV
							(a × b)
Novelty seeking	Reappraisal	Positive emotions	0.411 ***	0.208 **	0.529 ***	0.444 ***	0.085 **
Novelty producing	Reappraisal	Positive emotions	0.251 **	0.207 **	0.511 ***	0.459 ***	0.052 **
Flexibility	Reappraisal	Positive emotions	0.432 ***	0.211 **	0.532 ***	0.441 ***	0.091 **
Engagement	Reappraisal	Positive emotions	0.227 **	0.233 **	0.462 ***	0.409 ***	0.053 **
Engagement	Suppression	Negative emotions	−0.298 ***	0.216 **	−0.495 ***	−0.431 ***	−0.064 **

Note. IV = independent variable; M = mediator; DV = dependent variable. Standardized coefficients are reported. ** *p* < 0.01. *** *p* < 0.001.

## Data Availability

The data presented in this study are available on request from the corresponding author.
